# Aesthetic Treatment of Bilateral Peg-Shaped Lateral Incisor and Restoration of a Harmonious Smile: A Case Report

**DOI:** 10.1155/crid/3371424

**Published:** 2025-07-08

**Authors:** Abdullah Alshehri

**Affiliations:** Conservative Dental Science Department, College of Dentistry, Prince Sattam Bin Abdulaziz University, Al-Kharj, Saudi Arabia

## Abstract

Peg-shaped lateral incisors are the most prevalent tooth size discrepancy, which can have a number of functional and aesthetic implications in affected people. This case report presents a conservative treatment approach to treat bilateral peg laterals and restore aesthetic harmony. A 21-year-old male patient complained about the shape and unaesthetic appearance of his two front teeth which negatively impacted his smile. The patient presented with bilateral peg laterals in the maxillary arch, and he had a stable occlusion with normal overjet and overlap and good periodontal health. Digital smile design (DSD) analysis revealed a discrepancy in crown width between Teeth #13 and #23 and gingival height discrepancy between Teeth #11 and #21, and #12 and #22. Accordingly, it was planned to perform a crown lengthening procedure to overcome the gingival height discrepancy of Teeth #12 and #21, place a full-contour resin composite restoration on Teeth #12 and # 22 and increase the mesiodistal width of Tooth #13. The patient underwent home teeth whitening for 2 weeks, followed by a 1-week observation period to ensure colour stability of the tooth. Laser gingivectomy was performed to correct the gingival height discrepancy, and incremental resin composite was used to restore the peg-shaped laterals. The restoration was contoured, finished and polished to obtain a smooth and glossy finish. The aesthetic treatment achieved excellent results, and the patient was pleased and excited with his new smile.

**Summary**

• Direct resin composite can be a favourable option compared to a more invasive and expensive treatment procedure for restoring peg-shaped laterals.
• The use of different resin composite restorative materials and techniques allows for a more practical and conservative treatment approach to treat peg-shaped laterals.

## 1. Introduction

In the present digital world, people of all ages are becoming increasingly more appreciative about their smile and physical appearance. The smile is amongst the most crucial aspects of people's perception and self-perception. This has a direct impact on people's facial expressions and physical appeal. Furthermore, positive traits such as intelligence, good health, sociability and assertiveness are all associated with a graceful and cordial smile [1, 2]. On the contrary, dental aberrations, particularly in the anterior region of the oral cavity, such as alteration in shape, dimension, position, number or colour, might cause aesthetic disharmony. A multitude of hereditary and environmental factors can cause dental abnormalities. Despite the fact that both the prenatal and postnatal stages are associated with dental aberrations, events during the perinatal stage have the greatest impact on these defects [3, 4].

The occurrence of conical tooth, also known as peg shaped, of upper lateral incisors (*herein after referred to as peg laterals*) is reported to be predominantly high than other morphological variations of permanent teeth [5, 6]. Peg laterals are linked to defects in some specific genes [7] and is characterised by considerable reduction in incisal mesiodistal dimension compared to cervical region [4, 8]. The shape and dimension of the peg laterals create anterior diastemas, which causes considerable functional and aesthetic challenges for affected individuals, in addition to creating a disharmonious smile that draws the viewer's gaze [1, 8]. The prevalence of peg laterals has ranged from 0.6% to 9.9% [9], with a slight female predominance (1:1.35) [5, 9]. They commonly occur on the left side of the arch compared to the right side (2:1), despite the fact that the incidence of unilateral and bilateral lateral incisors is the same [9].

Peg laterals are a major concern for general dentists as well as dental specialists, since they can cause aesthetic, orthodontic and periodontal problems for those who are affected [8]. Treatment options for peg laterals are determined by the patient's expectations as well as the clinician's skill [10]. The most exorbitant treatment option is the extraction of the peg lateral. However, this is best followed if there is an insufficient root support or a deformed root; the extraction of the offended tooth could pave the way for tooth replacement with an implant, adjacent tooth preparation for a fixed partial denture (FPD) and/or orthodontic movement of the canines into the extraction space. Contrarily, a peg lateral with sound root could be treated by a full crown, indirect ceramic veneers or a more conservative direct composite veneers [8, 10].

The aim of this case report is to present a conservative, comparatively inexpensive treatment protocol to treat bilateral peg laterals and restore aesthetic harmony using direct composite bonding.

## 2. Case Presentation and Treatment Planning

**Figure 1:**
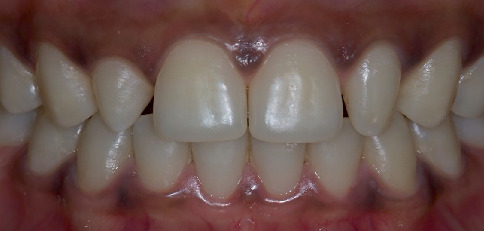
Smile view displaying the upper and lower anterior teeth.

A 21-year-old male patient of African origin was referred to restorative dental sciences department at King Saud University. The patient's main complaint was about the shape and unaesthetic appearance of his two front teeth which negatively impacted his smile. The patient's medical history was nonrelevant, and dental history included oral prophylaxis and few posterior resin composite restoration. On intraoral clinical examination, the patient presented with bilateral peg lateral in the maxillary arch. The patient had a stable occlusion with normal overjet and overlap and good periodontal health (Figure 1). Further examination complemented by radiographs revealed acceptable and healthy tooth structure, adequate bone support and the lack of periapical diseases. The clinician responded positively to patient's queries and also explained about the available treatment options. The patient had high aesthetic expectations regarding his tooth shape and smile, and the time available for the treatment was limited since he was travelling abroad. Hence, it was agreed upon to treat the peg laterals by direct resin composite bonding.

**Figure 2:**
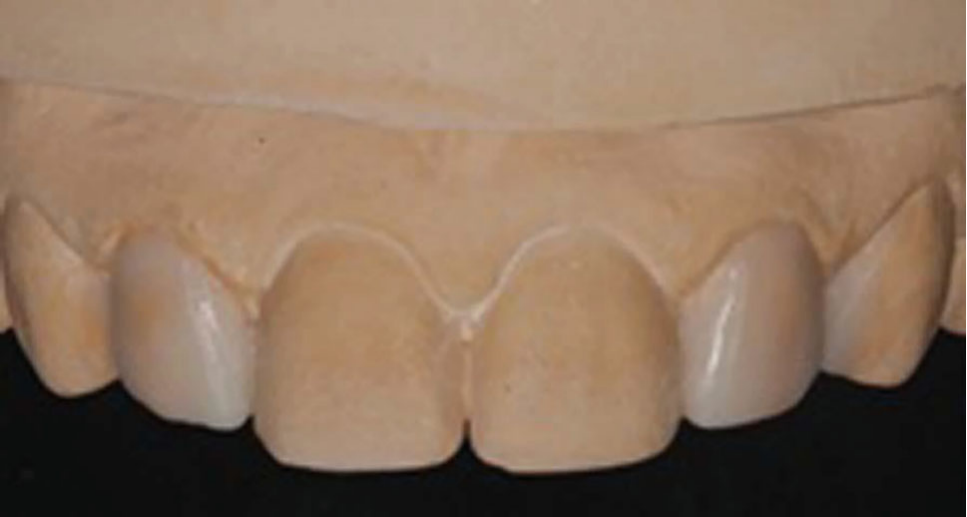
Diagnostic wax-up of the upper lateral incisors.

During the same appointment, alginate impression of maxillary and mandibular arch was made to obtain study casts, and the patient was scheduled for his next visit after 3 days. Digital smile design (DSD) analysis was performed to assess facial harmony, optimal tooth aesthetics and dentogingival characteristics [11, 12]. The outcome of the DSD analysis revealed discrepancy in crown width between Teeth #13 and #23 and gingival height discrepancy between Teeth #11 and #21, and #12 and #22 (Table 1). Based on the outcome of the DSD analysis, it was planned to perform a crown lengthening procedure by gingivectomy to overcome the gingival height discrepancy of Teeth #12 and #21, place a full-contour resin composite restoration on Teeth #12 and # 22 and increase the mesiodistal width of Tooth #13 by adding resin composite to the mesial side. A wax setup was fabricated in the study cast to simulate the posttreatment tooth form and design of peg laterals. The wax setup also assesses the patient's perception of the recommended treatment plan and facilitate the manipulation of the final restoration (Figure 2). The patient was informed about the treatment plan based on the DSD and wax setup, and after visualising the mock-ups and the predictability of the final outcome, the patient consented to undergo the treatment.

## 3. Clinical Procedure

### 3.1. Home Teeth Whitening

**Figure 3:**
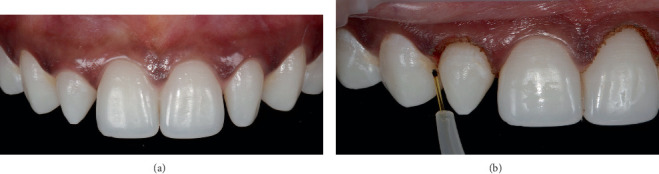
(a, b) Aesthetic gingivectomy was performed to overcome the gingival height discrepancy on Teeth #12 and #21 using a soft tissue diode laser.

Resin composite restoration complemented by teeth whitening enhances the outcome of the restorative treatment and the final smile. The patient was instructed to use home whitening strips (Crest 3D Whitestrips, Procter & Gamble, OH, United States) for 30 min daily for 2 weeks. A 1-week waiting period was maintained between the completion of whitening and commencement of crown lengthening (Figure 3).

### 3.2. Gingivectomy

Aesthetic crown lengthening was performed to overcome the gingival height discrepancy on Teeth #12 and #21 using a soft tissue diode laser (Den-Mat Holdings, LLC, CA, United States) with a wavelength of 808 nm (±5 nm). The laser was set to work on the continuous wave mode at 1.4 W and configured with an initiated tip, and gingivectomy was performed using the contact technique. The patient had mild pain with uneventful bleeding (Figure 3b).

### 3.3. Direct Resin Composite Restoration

**Figure 4:**
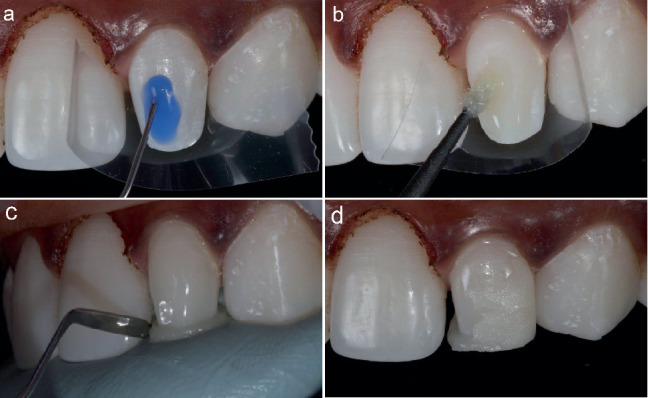
(a–d) 35% phosphoric acid gel, adhesive application and the placement of dental composite.

A lingual silicone index was fabricated using the diagnostic wax-up to precisely determine the palatal contour and incisal surface of the peg laterals. After isolating the adjacent teeth using a Mylar strip, the tooth to be restored was etched with 35% phosphoric acid gel (Kerr Gel Etchant; Kerr, Orange, CA, United States) for 30 s (Figure 4a). The tooth was washed off the etchant using an air–water syringe that revealed a white chalky surface. A single coat of dental adhesive (OptiBond Universal, Kerr, Kloten, Switzerland) was applied onto the etched enamel surface and light cured for 20 s using an LED light curing unit (Elipar DeepCure-S, 3M ESPE, St. Paul, MN, United States) (Figure 4b).

Initially, the restoration was built using effect enamel light cure resin composite (ESTELITE OMEGA, Shade MW, Tokuyama Dental Corporation, Tokyo, Japan) with the silicone index as a guide and light cured for 20 s. Next, an increment layer was built over the base resin composite using the light cure microfilled resin composite (Durafill VS, Shade SSL, Heraeus Kulzer GmbH, Hanau, Germany) and light cured for 20 s (Figure 4c,d). The restoration was then checked for any occlusal adjustment before finishing and polishing.

**Figure 5:**
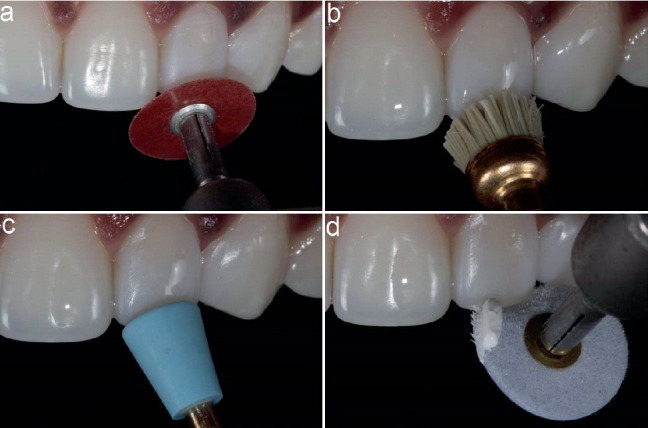
(a–d) Finishing and polishing sequence using various selection of discs and cups.

**Figure 6:**
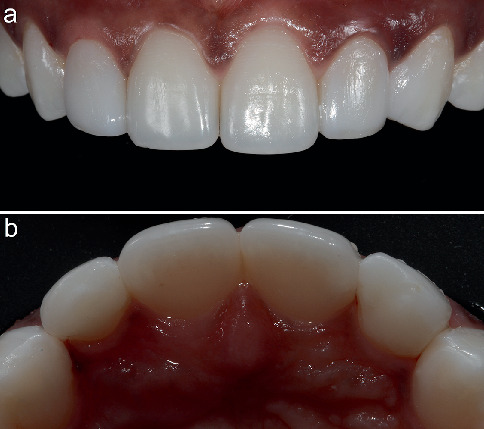
(a, b) Final restoration intraoral.

**Figure 7:**
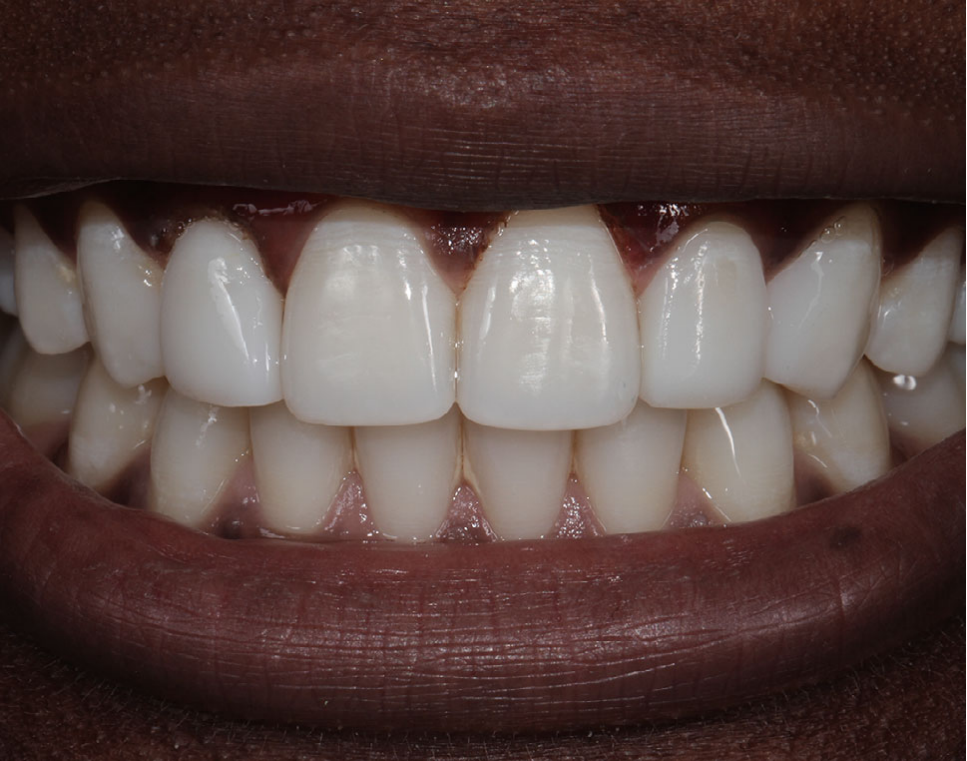
Final restoration smile view.

The composite restoration was contoured and finished using aluminium oxide–coated discs (Sof-Lex discs; 3M ESPE, St. Paul, MN, United States) and rubber bristles and cups (Jiffy, Ultradent, South Jordan, UT, United States). Finally, the restoration was polished using flexi buffs and polishing paste (Enamelize, Cosmedent, Chicago, IL, United States) (Figures 5a, 5b, 5c and 5d). The patient was pleased and excited with the treatment outcome and his new smile. The posttreatment images of labial and palatal view of the peg laterals are presented in Figure 6a,b. The patient's new smile is presented in Figure 7.

The patient was recalled after 1 week to evaluate the final shade of the resin composite. The restoration was intact, and there was no marginal staining. The patient was emphasised about good oral hygiene maintenance and future follow-ups to assess the integrity of the restoration. The patient was lost to further follow-up as he had travelled abroad for higher studies.

## 4. Discussion

An individual's facial aesthetics, conscience and contentment are all influenced by their smile [13]. The teeth, which are framed by the lips, the gingival contour, the amount of spacing and tooth form and shape, all contribute to the smile aesthetics [14, 15]. Since a harmonious smile is the consequence of the interaction of a variety of factors of different importance, an understanding of the treatment concepts that establish the balance between dental practitioners' knowledge of smile aesthetics and patients' perceptions is critical [16].

To achieve the desired aesthetic outcome, ideal sequencing and planning should be taken into consideration; the tooth whitening protocol prior to placement of composite aided in unifying the colour of the teeth, resulting in easier and better shade matching that eventually enhanced the final treatment outcome.

With different tooth substrates (i.e. enamel and dentin), choosing the correct composite composition is crucial to match the characteristics of the tooth substrate to be replaced. Composite layering is used to mimic the natural appearance of the tooth enamel and dentin, as it allows for customisable colour, translucency and texture, ultimately improving both the aesthetic outcome and the longevity of the restoration.

DSD is changing the way dentists plan treatments by making the process more personalised, precise and collaborative. Instead of relying only on traditional tools like physical moulds or static photos, DSD uses digital images, videos and advanced software to design a smile that truly fits a person's face, expression and personality. It lets both the dentist and the patient see a preview of the final result before any work begins, which helps build trust and clarity. With DSD, treatment planning becomes more than just a clinical process—it becomes a creative, patient-centred journey towards a smile that feels as good as it looks [17].

Diode laser gingivectomy has gained increasing attention as a minimally invasive alternative to conventional scalpel and electrosurgery techniques, offering favourable clinical outcomes and improved patient experience. Unlike the scalpel, which often necessitates suturing and results in increased intraoperative bleeding, the diode laser provides precise soft tissue ablation with effective coagulation, thereby enhancing surgical visibility and reducing the need for haemostatic interventions. Compared to electrosurgery, diode lasers generate less lateral heat, minimising thermal damage to adjacent tissues and promoting more predictable healing. Numerous studies have demonstrated that diode laser gingivectomy is associated with reduced postoperative pain, swelling and discomfort, along with shorter healing times and a lower risk of complications. These benefits contribute to higher patient satisfaction and improve clinical efficiency, supporting its integration into contemporary periodontal and aesthetic treatment protocols [18–20].

Peg-shaped laterals are crucial to diagnose and manage in clinical practice because they are frequently associated with aesthetic and functional clinical problems. For a predictable outcome, appropriate diagnosis, adequate aesthetic evaluation and careful consideration of treatment alternatives are recommended. In the current case, a conservative treatment approach was established to treat bilateral peg laterals and restore aesthetic harmony. A diode laser was used to accomplish the crown lengthening to overcome the gingival height discrepancy of Teeth #12 and #21, and in the same visit, resin composite restoration was performed to restore the form of peg laterals. In recent years, lasers have been widely employed for gingivoplasty and gingivectomy procedures in traditional dental therapy. When compared to the conventional scalpel technique, the soft tissue treatments using diode laser have several advantages such as uncomplicated tissue ablation, homeostasis, antibacterial effects, reduced pain and discomfort and no or minimal suturing [13, 21–23].

Full-contour resin composite restoration was performed on Teeth #12 and #22, and the mesiodistal width of Tooth #13 was extended by adding resin composite to the mesial surface. Peg-shaped laterals restored with resin composites is regarded as the most conservative treatment option and presents with numerous advantages such as preservation of sound tooth structure, optimal aesthetics, reduced treatment time and cost-effectiveness [24]. Furthermore, the restorations are easily added, contoured, finished and polished to mimic a natural tooth [25, 26]. Tooth whitening in adjunct to resin composite restoration also greatly enhances clinicians' ability to preserve the remaining healthy tooth structure.

The patient was recalled after 1 week to review the colour stability of the final resin composite. Since the treatment is performed on isolated teeth that have been dehydrated, the composite shade chosen may be too light. As a result, it is crucial to inspect the restoration, and this gives a chance for the clinician to smoothen any rough edges and repolish, as well as affirm the functional occlusion. The patient had high aesthetic expectations before the treatment; however, following treatment, he was very happy with his smile and the shape of his anterior teeth. The proposed protocol used here is technique sensitive and requires practice; clinicians need to practice this technique and master it to be able to integrate it in their daily routine.

A study by Montinaro et al. [27] concluded that the normal symmetrical appearance of the central incisor, lateral incisor and canine had the best aesthetic results for all subjects. Thus, pig lateral treatment planning is challenging; one of the most conservative approaches is dental composites, where composite resin is applied to reshape and enhance the peg-shaped tooth. This technique offers a relatively quick solution that can be completed in a single appointment, promoting immediate aesthetic improvement whilst allowing for reversibility and minimal invasiveness [28]. However, the longevity and resistance to wear of bonded materials must be considered, as they may require periodic replacement or repair [29].

Porcelain veneers represent another effective option for patients seeking durable aesthetic enhancements. These custom-made ceramic shells are bonded to the facial surface of the tooth, providing improved aesthetics through their superior translucency and colour stability [30]. Although the procedure necessitates the removal of a small amount of tooth structure, the long-term aesthetic and functional benefits often justify this approach [31].

Composite crowns also serve as a viable solution, especially in instances where significant structural alteration is necessary. By covering and reinforcing the existing tooth, composite crowns can enhance both aesthetics and functional capabilities [32].

In summary, the management of peg-shaped laterals necessitates a multifaceted approach that considers individual patient needs, aesthetic goals and clinical conditions. An interdisciplinary evaluation and treatment planning by dental professionals are crucial for determining the most appropriate and effective treatment modality, ultimately enhancing oral health and quality of life for affected individuals.

## 5. Conclusion

The preservation of natural tooth structure with adhesive restorations is one of the most important developments in contemporary restorative dentistry. Clinicians should take advantage of new techniques and materials to minimise the need for more extensive restorative treatment on a regular schedule and defer costly crown and bridge and implant treatments. The use of resin composites and laser gingivectomy to restore peg-shaped laterals and a harmonious smile in the present case demonstrated excellent treatment results, and the patient was pleased and excited with his new smile.

## Figures and Tables

**Table 1:** Maxillary anterior tooth dimension according to digital smile design (DSD) and notes regarding the resin composite restoration.

**Tooth No.**	**Width (in mm)**	**Length (in mm)**	**Notes**
13	6	8	Increase mesiodistal width by adding to the mesial side
12	5	6	Full-contour composite restoration
11	7	9	No modification required
21	7	8.5	No modification required
22	5	7	Full-contour composite restoration
23	7	8	No modification required

## Data Availability

Data sharing is not applicable to this article as no datasets were generated or analysed during the current study.
